# American Dental Convention

**Published:** 1857-10

**Authors:** 


### EDITORIAL DEPARTMENT.

*American Dental Convention.*—A full report of the proceedings of the last meeting of the American Dental Convention will be found in the present number of the Journal, taken by a stenographer, employed for the occasion, by Dr. J. R. McCurdy, one of the editors of the Dental News Letter, and for his courtesy in promptly furnishing us with a copy, we beg to tender him our most grateful acknowledgments.

The Convention, as we anticipated before the meeting, was numerously attended; twenty-three states of the Union were represented. The number of Dentists present, was supposed to be upwards of two hundred. The Secretary, however, was not able to obtain the names of all who were there. The entire meeting was characterized by the utmost harmony of feeling, and most of the discussions were highly interesting.

The cordiality with which the members of the profession from abroad, were received by their Boston brethren, will ever be remembered with feelings of the liveliest satisfaction. On the afternoon of the third day they were invited by the dentists of Boston and vicinity, to an entertainment on board the steamer Neptune. Accordingly, at 1½ o'clock, the members proceeded in a body to the end of Long Wharf, where they embarked, the steamer being in waiting to take the party on board. The senior editor, expecting to leave Boston by the afternoon cars, was prevented from participating in the festivities of the occasion. It was, as we afterwards learned from several who were present, a very splendid affair. The Boston Bee, of the next morning, gives the following description of the excursion and entertainment:

Shortly after leaving the wharf, a fish chowder prepared in Smith's best style, was served, greatly to the satisfaction of keen appetites.

Dr. E. T. Wilson, of this city, who acted as chairman of the occasion, took the platform and welcomed his brethren as follows:

In behalf of the Dentists of Boston and vicinity, I am happy to welcome the representatives of the dental profession from the North, East, South, and

West, to this social meeting. I did hope the duty would devolve upon one of two individuals, who have largely contributed to the present elevated position of the profession, not only in this country, but throughout the world. I allude to those distinguished men, Dr. Harwood and Dr. Joseph Tucker, of Boston, to whom we owe a heavy debt of obligation, and to pay it best we must follow in their footsteps. They have been strong pioneers, who have broken the rough soil and built the smooth road. We must follow on their track, and benefiting by their experience, be filled with their ambition. When we do this, there will be no longer a doubt about our claim to position amongst the members of the most liberal professions—we shall be regarded not only as men of mechanical skill, but as wise and large contributors to the health, the happiness, and the comfort of our fellow men. Gentlemen, I again bid you hearty welcome.

Dr. Townsend, of Philadelphia, was called upon to respond, which he did in a brief manner. He thought it hardly fair, after they had been invited to a feast, that they should have anything *extracted* from them, particularly at so early a period of the excursion. He begged, however, in behalf of those who were recipient of the generous courtesies bestowed upon them, to return his and their sincere thanks.

The company were then invited to partake of a collation. The invitation was accepted, and due justice was done to the spread, both viand and vinous.

An hour or two was spent sailing about and below the harbor, when the Neptune proceeded towards Nahant. When some four miles off that renowned peninsula, the boat came to a stop, when an *attempt* was made to catch some fish. This was, however, much to the disappointment of many, quite unsuccessful. Not a fish—only two sculpins were caught. Considering that nearly every man on board obtains his living by drawing, their ill luck may be put down in the book of marvels.

Hauling up lines, the steamer proceeded to Nahant, where an hour or more was spent in roaming about the peninsula, chatting at the Nahant House, and various amusements. On landing, the company formed in procession, and marched, led off by Bond's Cornet Band, which accompanied the excursion, in line to the hotel. While here, the band performed several popular pieces much to the gratification of all.

Leaving Nahant at 6 o'clock, the Neptune wended her way, by a circuitous and most pleasant route, to Hull, where the company again formed in procession, and marched to the Mansion House. While here the excursionists were the recipients of munificent hospitalities from Dr. E. G. Tucker, the newly elected Vice-president of the Convention. Short and *brusque* speeches were made at the tables. Subsequently, outside the hotel, a large circle was formed, when Dr. Tucker, apologizing for lack of oratorical proficiency, introduced John C. Wyman, Esq., who welcomed the members to Boston, and also to Hull. His speech was quite eloquent, appropria[illegible] and happy, and was received with applause. Other speeches were made by Dr. Tucker, and Dr. Spaulding, of Dedham,

Leaving Hull amid the cheers of the natives who lined the wharf, and the waving of handkerchiefs by the ladies, the Neptune took her course homeward. While on the passage up, Dr. Wilson took the platform and introduced several gentlemen, who made speeches. Among them were the following: Prof. Taylor of Cincinnati, President of the Convention; Drs. Dwinelle of New York, Townsend of Philadelphia, Allport of Chicago, Hitchcock of Boston, who made a most eloquent effort; Kendrick of Natchez, Miss., Forbes of St. Louis, Rogers of Utica, N. Y., Taft of Ohio, J. A. Cummings of Boston, Dalrymple of New York, Perkins of Wis., Perine of New York, Buckingham of New York, Fuller of Portsmouth, N. H., Atkinson of Cleveland, Ohio, Post of Vermont, Allen of New York, B. S. Codman of Boston, Coates of Virginia, McCurdy of Philadelphia, Hamlin of Tenn., Ball of Boston, Carrie of Maine, and others. The speeches were of an unusually good stamp, and elicited hearty applause. We regret that the crowded state of our columns does not permit even a running sketch.

While on the homeward trip, Dr. Fuller, of Portsmouth, N. H., offered a resolution of thanks to the dentists of Boston and vicinity, for their courtesies to the members of the profession from abroad. The resolution was introduced with a highly complimentary speech, and was most cordially and unanimously adopted.

The boat reached Long wharf shortly before 10 o'clock, when the company again formed in procession, and marched, to a lively tune, to the Tremont House, where a season of festivity was renewed. At a late hour they separated, highly and heartily gratified with Boston hospitality.

The excursion was in every respect a pleasant one. The weather was favorable, the harbor smooth, the refreshments good, and everything passed off, so far as we are aware, to the satisfaction of all. The gentlemen for whose pleasure it was projected, expressed their cordial gratification. We trust that it may hold an agreeable place in their memory.

In this connection, while paying the tribute of an earnest compliment to the Committee of Arrangements for their successful labors, we beg to mention the very efficient services of Dr. Wm. P. Leavitt, and Mr. J. T. Freeman, connected with the dental depot of Jones, White & McCurdy. These gentlemen devoted their time to the heavy drudgery necessary to be done by *somebody* on all such occasions. Our readers well know that when Dr. Leavitt puts his hand to a work, it is carried on to success.

We have always been of the opinion, that the *fillings* of the Boston dentists never come out, but, as would appear from the above, it would seem they are not quite as skillful as we had before supposed. Many of the *fillings* put in on this occasion, as we were credibly informed, remained in but for a short time. This, however, may have been owing to the hasty manner in which the operations were performed, or to redundancy of material—for as to excellency in quality, all we have heard speak of the matter, bear the most ample testimony. That too much *moisture* had anything to do with the failures, we cannot for a moment suppose, knowing, as all dentists do, that *dryness,* especially in the use of the spongy or plastic material, is regarded as essential to a good *filling.* We would not, therefore, on any account, make so improbable an insinuation. It may be the *fillings* were only designed to subserve a temporary purpose, or the fault, if any, may have been as much with the *operatees* as with the *operators,* or, it is possible the former assumed the capacity of the latter, and if this were the case, as we shrewdly suspect it was, the insecurity of the operations may have been owing to the fact that expedition, rather than excellency of execution, was the point chiefly aimed at.

The next meeting of the Convention, as will be perceived by the proceedings of the last, is to be held at Cincinnati, Ohio. That it will be well attended, we think we are safe in predicting.

